# True versus pseudo-occlusion of the cervical internal carotid artery in acute stroke: A multicenter MR angiography study

**DOI:** 10.1093/esj/23969873251355450

**Published:** 2026-01-01

**Authors:** Christian Heitkamp, Pia Niederau, Arndt-Hendrik Schievelkamp, Nikolaos Ntoulias, Lukas Goertz, David Zopfs, Kai R Laukamp, Thomas Schömig, Jonathan Kottlors, Christian Nelles, Simon Lennartz, Marios-Nikos Psychogios, Franziska Dorn, Uta Hanning, Jens Fiehler, Michael Schönfeld

**Affiliations:** Department of Neuroradiology, University Medical Center Hamburg-Eppendorf, Hamburg, Germany; Department of Radiology and Neuroradiology, University Hospital Cologne, Cologne, Germany; Department of Radiology and Neuroradiology, Lucerne Cantonal Hospital, Lucerne, Switzerland; Department of Radiology and Neuroradiology, University Hospital Cologne, Cologne, Germany; Department of Neuroradiology, University Hospital Basel, Basel, Switzerland; Department of Radiology and Neuroradiology, University Hospital Cologne, Cologne, Germany; Department of Radiology and Neuroradiology, University Hospital Cologne, Cologne, Germany; Department of Radiology and Neuroradiology, University Hospital Cologne, Cologne, Germany; Department of Radiology and Neuroradiology, University Hospital Cologne, Cologne, Germany; Department of Radiology and Neuroradiology, University Hospital Cologne, Cologne, Germany; Department of Radiology and Neuroradiology, University Hospital Cologne, Cologne, Germany; Department of Radiology and Neuroradiology, University Hospital Cologne, Cologne, Germany; Department of Neuroradiology, University Hospital Basel, Basel, Switzerland; Department of Neuroradiology, University Hospital Bonn, Bonn, Germany; Department of Neuroradiology, University Medical Center Hamburg-Eppendorf, Hamburg, Germany; Department of Neuroradiology, University Medical Center Hamburg-Eppendorf, Hamburg, Germany; Department of Neuroradiology, University Medical Center Hamburg-Eppendorf, Hamburg, Germany; Department of Radiology and Neuroradiology, University Hospital Cologne, Cologne, Germany

**Keywords:** Stroke, thrombectomy, MR angiography, digital subtraction angiography%

## Abstract

**Introduction:**

Differentiating true from pseudo-occlusion of the cervical internal carotid artery (ICA) in acute ischemic stroke patients undergoing thrombectomy is crucial but challenging. We aimed to investigate the ability of contrast-enhanced magnetic resonance angiography (CE-MRA) to differentiate true from pseudo-occlusion (defined as an isolated thrombus of the intracranial ICA suppressing ascending blood flow) of the cervical ICA in acute ischemic stroke patients.

**Materials and methods:**

Multicenter, retrospective analysis of acute ischemic stroke patients with true or pseudo-occlusion of the cervical ICA and subsequent thrombectomy. Patients with preprocedural CE-MRA showing a lack of contrast filling in the cervical ICA on the symptomatic side were included. Six readers (three radiology fellows and three board-certified radiologists) independently evaluated the CE-MRA images for true or pseudo-occlusion of the cervical ICA using a rating scheme. Their assessments were compared with DSA results as the reference standard. Diagnostic accuracy measures, as well as inter- and intra-reader reliability for detecting pseudo-occlusion, were calculated and compared between subgroups.

**Results:**

A total of 41 patients were included. The median age was 73 years, and 39% were female. According to the reference standard, 16 of 41 (39%) patients had a pseudo-occlusion of the cervical ICA, while the remainder had a true occlusion. The aggregated sensitivity and specificity from all readers were 72% (95% confidence interval [CI]: 62%–81%) and 86% (95% CI: 79%–91%), respectively. Board-certified radiologists performed better, with a sensitivity of 81% (95% CI: 67%–91%) and specificity of 92% (95% CI: 83%–97%). Overall, inter-reader agreement was moderate (κ = 0.48; 95% CI: 0.31–0.65) and reached substantial agreement within the board-certified radiologists subgroup (κ = 0.65; 95% CI: 0.45–0.85).

**Conclusion:**

Differentiating true occlusion from pseudo-occlusion of the cervical ICA using CE-MRA is feasible but requires training in specific imaging characteristics as well as experience in interpreting them, as evidenced by the higher diagnostic accuracy of board-certified radiologists. Correct distinction help in optimal material selection (e.g. size and type of guiding catheter) prior to endovascular treatment.

## Graphical Abstract

**Graphical abstract:**
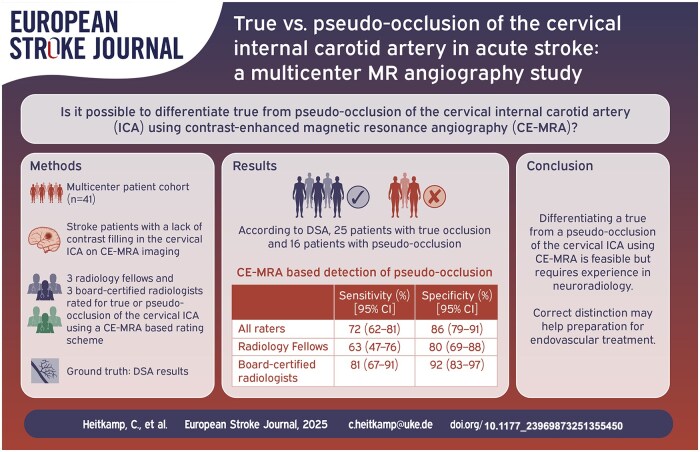

## Introduction

Acute ischemic stroke (AIS) due to large vessel occlusion (LVO) represents a significant burden on healthcare systems worldwide, necessitating rapid and accurate diagnostic strategies for optimal therapeutic management.^1,2^ Among the diverse etiologies of AIS, cervical internal carotid artery (ICA) occlusions present a diagnostic challenge,^3,4^ particularly in distinguishing between true occlusions from pseudo-occlusions, characterized by a flow artifact caused by an isolated thrombus of the intracranial ICA that suppresses ascending blood and consequently contrast flow without complete occlusion of the cervical ICA.^5^ Differentiation between these entities is crucial for appropriate endovascular treatment decisions, as pseudo-occlusions may necessitate different technical approaches than true occlusions, for example, optimal choice of catheter system prior to mechanical thrombectomy (MT).

To date, previous investigations have primarily focused on CT angiography (CTA) and concluded that CTA is an unreliable modality for distinguishing true occlusions from pseudo-occlusions.^5^ During CTA, a rapid arterial phase may “outrun” the delayed arrival of contrast medium in a patient with an isolated intracranial thrombus, leading to the false assumption of a true occlusion of the cervical ICA. We hypothesized that MRI contrast agents would remain detectable in the vessel for a longer period, making visibility less time-sensitive with regards to bolus timing. From a practical perspective, MRI is frequently used as the primary imaging modality in AIS-LVO patients, as it offers higher sensitivity in detecting areas of ischemia and enhanced diagnostic precision in differentiating ischemic strokes from stroke-mimics.^6,7^ Yet, its capability to accurately differentiate true from pseudo-occlusion of the cervical ICA remains understudied.

In this multicenter study of AIS-LVO patients who underwent MT, we aimed to investigate the diagnostic potential of CE-MRA in distinguishing between true and pseudo-occlusion of the cervical ICA by using a rating scheme that has been further developed on the basis of CTA-based rating schemes.^8–10^ We hypothesized that a distinction between true and pseudo-occlusion is possible using CE-MRA. This may help to improve clinical-decision making in AIS-LVO patients prior to endovascular therapy.

## Methods

### Patients and study design

This retrospective study was conducted in accordance with the Guidelines for Reporting Reliability and Agreement Studies (GRRAS)^11^ and the Standards for Reporting of Diagnostic Accuracy Studies (STARD).^12^ Our study comprises data from four distinct European stroke centers (University Hospital Cologne, Germany; University Medical Center Hamburg-Eppendorf, Germany; University Hospital Bonn, Germany; The University Hospital Basel, Switzerland). The inclusion period encompasses the years 2015–2021. Acute ischemic stroke patients whose CE-MRA revealed a lack of contrast filling in the cervical ICA on the symptomatic side and who then underwent mechanical thrombectomy with or without prior intravenous tissue plasminogen activator therapy were included in our study. The assessments of these cases were performed by local board-certified radiologists as part of clinical routine and were subsequently confirmed by DSA results during MT. Exclusion criteria encompassed patients with trauma, posterior circulation stroke, and those lacking adequate preprocedural CE-MRA imaging quality. Due to the retrospective nature of the study, informed consent was waived by the local ethics committee of each participating center. Supplemental Figure S1 illustrates a comprehensive flow diagram of all included patients.

### Imaging and ratings: Classification and categories

The CE-MRA images were acquired at the respective centers at which patients were treated by mechanical thrombectomy. CE-MRA was performed in the setting of acute ischemic stroke symptoms prior to endovascular treatment. The imaging protocol for each patient included at least one arterial phase contrast-enhanced CE-MRA examination of the head and neck prior to endovascular treatment at all centers. The CE-MRA parameters and technology settings were comparable across all participating centers. Details on the MRI scanners and sequence parameters can be found in Supplemental Table S1.

**Figure 1.**
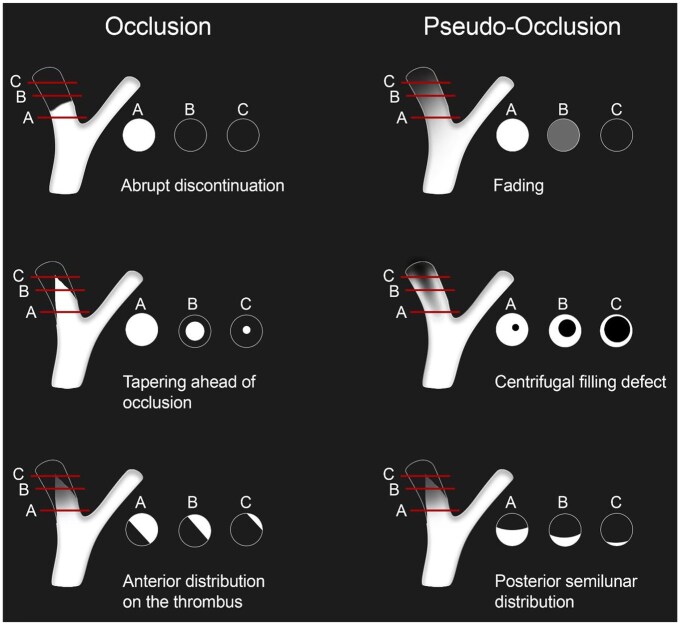
Schematic illustration depicting different imaging patterns related to the existence of a pseudo-occlusion and a true occlusion. Prior to and throughout the rating process, the raters were provided with this diagram and were instructed to indicate which one most accurately represented the case at hand.

Anonymized source images and multiplanar reformations of the arterial phase contrast-enhanced CE-MRA were provided to the raters using the local Picture Archiving and Communication System (PACS). Imaging data were independently assessed by six raters, encompassing three radiology fellows (L.G., J.K., T.S. with 3, 4, and 3 years of working experience in neuroimaging) and three board-certified radiologists (D.Z., K.L., C.N with 7, 7, and 6 years of working experience in neuroimaging). Based on the CE-MRA images, the raters were asked to determine the presence of a true or pseudo-occlusion of the cervical ICA and how confident they felt about their decision on a scale of 1–3 (1: uncertain, 2: intermediate, 3: certain). Prior to and during the ratings, the readers were provided with a rating scheme depicting different imaging patterns related to the existence of a pseudo-occlusion and a true occlusion (Figure 1). Raters were also asked to indicate the imaging pattern that best present the specific type of occlusion. The rating scheme was developed using prior research on CTA-based imaging patterns to distinguish pseudo-occlusions from true occlusions.^8–10^ All raters were blinded to any clinical information as well as the final DSA diagnosis.

According to a previous study,^5^ we identified the three raters that rated best with respect to the reference standard and asked them to review the same patients a second time to scrutinize their consistency and verify that they can preserve this high level of accuracy a second time. These ratings were performed at least 6 weeks after the first ratings to minimize recall bias.

### Reference standard

The results of the DSA in the context of mechanical thrombectomy were considered the reference standard. True occlusion of the cervical ICA was characterized by the presence of an obstruction in the vessel preventing passage of contrast agent and/or guidewire catheter during DSA. Additionally, if microcatheter passage was performed, the absence of backfilling in the cervical ICA during microcatheter angiography had to be present.^5,8^ According to a previous study,^5^ pseudo-occlusion was defined as an absence of a true cervical ICA occlusion in DSA, but a lack of contrast filling in the cervical ICA on CE-MRA imaging. The occlusion type taken from the report of the treating neurointerventionalist at the respective stroke center was confirmed by one of the authors (M.S., with 14 years of working experience as an interventional neuroradiologist).

### Statistical analysis

Sensitivity, specificity and accuracy rates pertaining to pseudo-occlusion were computed for each reader by using 2 × 2 contingency tables. Diagnostic accuracy measures for all readers, as well as the subgroups of fellows and board-certified radiologists, were calculated using the mean sensitivity, specificity, and accuracy values. The inter-reader and intra-reader agreement with respect to the occlusion type as a dichotomized outcome was assessed using Cohen’s Kappa for pairwise raters and Fleiss’ Kappa for more than two raters. The inter-reader agreement for the imaging pattern that best present the occlusion type was calculated using Krippendorff’s α. The level of agreement was interpreted according to Landis and Koch^13^ (slight agreement 0–0.2, fair agreement 0.21–0.4, moderate agreement 0.41–0.6, substantial agreement 0.61–0.8, and almost perfect agreement 0.81–1.0). The data analysis was conducted using Stata (Stata/MP18, StataCorp, TX, USA).

### Data availability

Data supporting the findings of this study are available from the corresponding author upon reasonable request.

## Results

### Patients’ baseline characteristics

Fourty one patients were included in the study (Supplemental Figure S1). The median age of included patients was 73 years (interquartile range: 59–81), with 39% being female. Pseudo-occlusion of the cervical ICA occurred in 16 of 41 (39%) patients and true occlusion in 25 of 41 (61%) patients.

### Diagnostic accuracy measures

**Figure 2.**
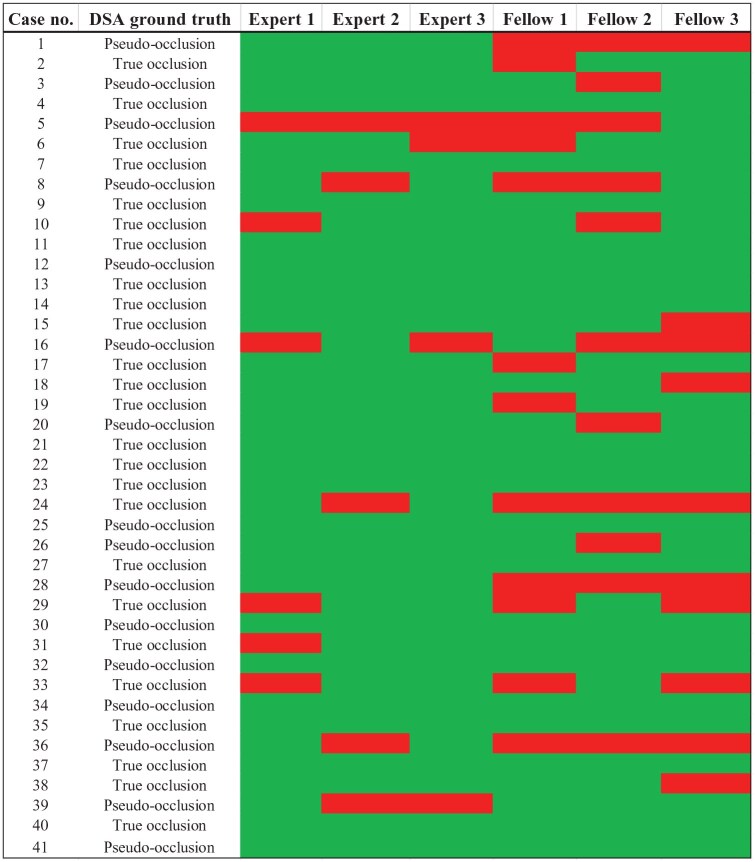
The results of all six raters are presented together with the digital subtraction angiography (DSA) as the ground truth. The green color represents a CE-MRA-based rating that is consistent with the occlusion type indicated by the DSA images, whereas the red color refers to an incorrect rating.

The individual sensitivity for detecting pseudo-occlusion ranged between 44% and 88%, while specificity varied between 72% and 96% (Supplemental Table S2). Figure 2 depicts the CE-MRA interpretations for all six raters together with the DSA results as the reference standard. Compared to fellow radiologists, the group of board-certified radiologists achieved higher point estimates of diagnostic accuracy values with a sensitivity of 81% and a specificity of 92%. The values increased even further when considering only those cases in which the board-certified radiologists reported a level of certainty of 2 and 3 (Table 1).

**Table 1.** Diagnostic accuracy measures and inter-reader agreement to differentiate between pseudo-occlusion or true occlusion of the cervical internal carotid artery.

Rater				Inter-reader reliability	
	Sensitivity (%)	Specificity (%)	Accuracy (%)	κ Value*	No. of raters
All (*n* = 246)	72 (62–81)	86 (79–91)	80 (75–85)	0.48 (0.31–0.65)	6
Board-certified radiologists (*n* = 123)	81 (67–91)	92 (83–97)	88 (81–93)	0.65 (0.45–0.85)	3
Certainty level^†^ ⩾ 2	83 (68–93)	95 (87–99)	91 (83–95)	0.89 (0.74–1.00)^a^	3
Certainty level^†^ = 3	88 (69–98)	97 (84–100)	93 (83–98)	1.00^b^	3
Fellows (*n* = 123)	63 (47–76)	80 (69–88)	73 (64–81)	0.44 (0.21–0.67)	3
Certainty level^†^ ⩾ 2	58 (41–73)	81 (69–90)	72 (62–80)	0.31 (−0.01 to 0.62)^c^	3
Certainty level^†^ = 3	50 (29–71)	89 (73–97)	73 (60–84)	0.07 (−0.38 to 0.53)^d^	3

Data in parentheses indicate 95% confidence intervals.

^a^Inter-reader reliability was calculated for 27 cases in which all three raters indicated a certainty level of at least 2.

^b^Inter-reader reliability was calculated for 5 cases in which all three raters indicated a certainty level of 3.

^c^Inter-reader reliability was calculated for 25 cases in which all three raters indicated a certainty level of at least 2.

^d^Inter-reader reliability was calculated for 7 cases in which all three raters indicated a certainty level of 3.

^*^Inter-reader reliability was calculated using Fleiss’ Kappa.

^†^Raters were asked to grade how confident they felt about their decision on a scale of 1–3 (1: uncertain, 2: intermediate, 3: certain).

### Inter-reader reliability

Pair-wise inter-reader agreements for the presence of true or pseudo-occlusion of the cervical ICA ranged from fair (κ = 0.35; 95% CI: 0.03–0.66) to substantial (κ = 0.72; 95% CI: 0.49–0.96). We observed a substantial agreement for our subgroup of board-certified radiologists (κ = 0.65; 95% CI: 0.45–0.85), while fellow radiologists reached only moderate agreement (κ = 0.44; 95% CI: 0.21–0.67). Consistent with the diagnostic accuracy measures, inter-reader agreement among board-certified radiologists increased when considering only cases for which raters indicated a certainty level of at least 2 or 3 (Table 1).

**Figure 3.**
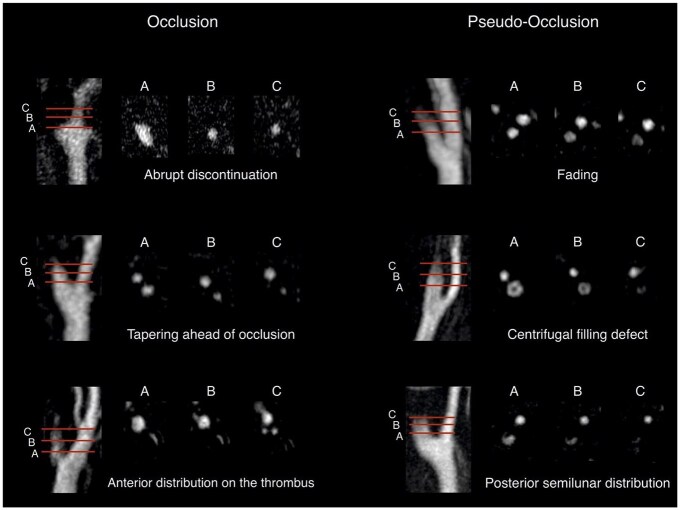
Axial reformations and maximum intensity projections (MIPs) of the arterial phase CE-MRA at the level of the carotid bifurcation in six patients. The images are aligned according to the schematic illustration in Figure 1, which depicts different imaging patterns associated with pseudo-occlusion and true occlusion. In all six patients, all six raters correctly classified the patterns as either pseudo-occlusion or true occlusion.

Regarding the imaging patterns that best present the specific occlusion type, our subgroup of board-certified radiologists (κ = 0.53; 95% CI: 0.39–0.66) reached moderate agreement, while fellows achieved fair agreement (κ = 0.36; 95% CI: 0.22–0.51). Figure 3 illustrates examples from 6 of the 19 patients for whom all raters achieved perfect agreement.

### Imaging patterns

The three imaging patterns most frequently determined by all raters were abrupt discontinuation (30%), tapering ahead of occlusion (23%) and posterior semilunar distribution (19%). The highest rate of correct ratings for a pseudo-occlusion was observed for the “posterior semilunar distribution” with 89% (41/46) for all raters and 100% (23/23) for board-certified radiologists. With regards to a true occlusion, the highest rate of correct ratings was found for “abrupt discontinuation” with 91% (67/74) correct responses for all raters. Although absolute percentages varied slightly between subgroups, “fading” yielded the lowest diagnostic accuracy for pseudo-occlusion, with correct identification in only 45% (10/22) of cases across all raters. Table 2 provides more detailed information about the percentage of correct ratings for each imaging pattern.

**Table 2.** The table presents the percentage of correct ratings for each imaging pattern.

Imaging pattern	All raters % (*n*)	Board-certified radiologists % (*n*)	Fellows % (*n*)	Best rater % (*n*)
Occlusion				
Abrupt discontinuation	91 (67/74)	92 (33/36)	89 (34/38)	92 (12/13)
Tapering ahead of occlusion	77 (44/57)	92 (24/26)	65 (20/31)	88 (7/8)
Anterior distribution on the thrombus	72 (18/25)	75 (12/16)	67 (6/9)	83 (5/6)
Pseudo-occlusion				
Fading	45 (10/22)	56 (5/9)	38 (5/13)	67 (2/3)
Centrifugal filling defect	82 (18/22)	85 (11/13)	78 (7/9)	100 (4/4)
Posterior semilunar distribution	89 (41/46)	100 (23/23)	78 (18/23)	100 (7/7)

The values in parentheses indicate the ratio between the number of times the respective imaging pattern was rated and the number of cases in which a true or pseudo-occlusion was actually present, as verified by the gold standard digital subtraction angiography (DSA). The best rater was defined as the one with the highest diagnostic accuracy in identifying pseudo-occlusion (see Figure 2, Rater 3).

### Intrareader variability

In the group of raters who exhibited the highest discriminatory ability between true and pseudo-occlusion, we observed substantial intra-reader agreement for two raters (κ = 0.70; 95% CI: 0.47–0.93 and κ = 0.78; 95% CI: 0.57–0.99) and almost perfect agreement for one rater (κ = 0.89; 95% CI: 0.73–1.00). Additionally, the second rating of the experts yielded slightly higher sensitivity (83%; 95% CI: 70%–93%), specificity (99%; 95% CI: 93%–100%) and accuracy (93%; 95% CI: 87%–97%) values than the first rating and the inter-reader reliability of experts increased to almost perfect agreement (κ = 0.85; 95% CI: 0.71–1.00).

## Discussion

In our multicenter retrospective study of AIS patients with a lack of contrast filling in the cervical ICA on CE-MRA imaging, diagnostic accuracy values for the differentiation between true and pseudo-occlusion were highly variable for individual raters. Inter-reader reliability was moderate for all raters, implicating that CE-MRA imaging may be used with caution for the differentiation between true and pseudo-occlusion for those with limited experience in radiology. However, our group of board-certified radiologists demonstrated superior discriminatory ability and achieved substantial to almost perfect inter- and intra-reader reliability, thereby indicating that a distinction is feasible for experienced radiologists.

In our study group, DSA confirmed pseudo-occlusion in 39% of AIS patients who presented with non-attenuation of the cervical ICA on the symptomatic side, which is in the range of previous studies.^5,10,14^ From a technical point of view, pseudo-occlusion occurs when a distal occlusion of the intracranial ICA causes sluggish or absent blood flow in the cervical ICA due to altered downstream hemodynamics. Rapid arterial phase during CTA may “outrun” the delayed arrival of contrast medium or contrast penetration in distal vessel segments is blocked by a stagnant column of unopacified blood.^10,14,15^ Previous studies have focused on CTA imaging and have suggested a number of imaging markers, including a mid-cervical flame shape stump and gradual contrast decline of the ICA,^9,10,16^ which may be beneficial in differentiating true from pseudo-occlusion. However, results with respect to the discriminatory ability were inconclusive.^5,8,10^ Recent studies have shown promising results using dynamic and multiphasic CTA, but this examination method is not yet widely adopted and is associated with an increased radiation exposure.^17–19^

On the other hand, MRI is frequently performed as the imaging modality of choice in selected patients with AIS who present with an unclear onset time (e.g. wake-up strokes) and minor or atypical symptoms. In this context, data regarding the detection of pseudo-occlusions with this imaging modality are scarce.

We therefore introduced CE-MRA-based imaging patterns, with which especially experienced board-certified radiologists were able to achieve high sensitivity and specificity values for differentiating between true and pseudo-occlusion. Imaging patterns indicating a pseudo-occlusion were a slow and tapered contrast decline above the level of the carotid bulb (“fading”), conically marginally tapering of contrast medium (“centrifugal filling defect”) or a dorsal semilunar distribution of contrast medium. Certain imaging features of these may serve as helpful indicators to differentiate pseudo-occlusion from true occlusion. In our study, “abrupt discontinuation” was most accurate for identifying true occlusion, while “posterior semilunar distribution” was the most reliable sign of pseudo-occlusion. The “fading” pattern should be interpreted with caution, as only 10 out of 22 cases were correctly identified as pseudo-occlusion, suggesting potential confusion with abrupt discontinuation. Focused attention to these patterns may support improved diagnostic accuracy. Interestingly, we observed that diagnostic accuracy values and inter-reader reliability improved with an increasing level of certainty for expert raters. It can therefore be assumed that experienced raters are highly proficient at evaluating the extent to which they can rely on the CE-MRA-based diagnosis of pseudo-occlusion. This finding has a significant impact in the clinical context of acute ischemic stroke treatment, as it is the experienced physician who is primarily responsible for the decision to perform MT. Identifying the correct type of ICA non-attenuation has a decisive impact on the choice of MT materials (e.g. choice of guiding sheaths, percutaneous transluminal angioplasty balloons, and distal access catheters).^5,10^ Choosing the right material from the start, may result in lower procedure costs, faster groin to recanalization times, fewer adverse events during treatment and ultimately improved functional outcomes.^5,10,14^ This is of particular importance in light of previous results which have demonstrated worse functional outcomes in patients with pseudo-occlusion.^14,17,20^

Limitations of this study include the retrospective study design and small patient group potentially introducing bias and minimizing the generalizability of our findings. Furthermore, all raters are affiliated with the same center, which may introduce bias. However, this limitation is mitigated by the fact that half of the included patients are from other centers. Unfortunately, our data do not incorporate information regarding the etiology of stroke, such as cardioembolic or large artery arteriosclerosis and the time duration between CE-MRA and DSA, including the first run from common carotid artery position. The possibility that patients were recanalized between CE-MRA and DSA due to the application of intravenous tissue plasminogen activator cannot be excluded, and this may have impacted the results of our ground truth measured by DSA. However, prior studies indicate that true cervical ICA occlusions tend to respond poorly to intravenous thrombolysis alone,^5,21^ and pseudo-occlusions that may have resolved would likely not have led to substantial changes in the ground truth.

## Conclusion

A CE-MRA-based differentiation between a true and a pseudo-occlusion of the cervical ICA in an acute ischemic stroke setting is feasible for experienced radiologists who have training in interpreting specific imaging characteristics. Our study further provides an easily applicable guideline of these distinct imaging patterns, which may contribute to a better differentiation. This could help to improve the choice of material for endovascular treatment, accelerate the procedure and thereby potentially improve patient outcomes.

## Supplementary Material

sj-docx-1-eso_23969873251355450

## Data Availability

Data supporting the findings of this study are available from the corresponding author upon reasonable request.

## Abbreviations

CE-MRA = Contrast-Enhanced Magnetic Resonance Angiography

ICA = Internal Carotid Artery

DSA = Digital Subtraction Angiography

AIS = Acute Ischemic Stroke

LVO = Large Vessel Occlusion

MT = Mechanical Thrombectomy
