# Supplementary material for: True versus pseudo-occlusion of the cervical internal carotid artery in acute stroke: A multicenter MR angiography study
Source: Eur Stroke J. 2026 Jan 1;11(1):23969873251355450. doi: 10.1093/esj/23969873251355450 (PMC12866277; doi:10.1093/esj/23969873251355450)
Supplement: sj-docx-1-eso_23969873251355450 [file sj-docx-1-eso_23969873251355450.docx]

Supplementary Material for

**True vs. Pseudo-Occlusion of the Cervical Internal Carotid Artery in Acute Stroke: A Multicenter MR Angiography Study**

Heitkamp et al.

**Figure S1**. Flow chart of patient inclusion and exclusion.

Table S1. Details on the MRI scanners and sequence parameters.

Table S2. Individual diagnostic accuracy measures.

**Figure S1**. Flow chart of patient inclusion and exclusion.

**Table S1.** Details on the MRI scanners and sequence parameters of the study centers.

| Study center | Scanner | Contrast | Flow | Parameters |
| --- | --- | --- | --- | --- |
| 1 | Philips Ingenia (3 Tesla) | Clariscan | Flowrate of 2 ml/s, succeeded by a 25-ml saline flush | CE-MRA Slice orientation Coronal Acquisition type 3D cartesian Acquired resolution (mm3) 0.63 × 0.63 × 0.63 Reconstructed resolution (mm3) 0.5 × 0.5 × 0.5 Field of view (mm3) 320 × 280 × 80 Flip angle 40° TR/TE (ms) 6.1/1.96 Nominal scan time (min:sec) 1:08 |
| 2 | Philips Achieva (3 Tesla) | Gadovist | Flowrate 1,5-3ml/s, succeeded by a 25-ml saline flush | CE-MRA Slice orientation Coronal Acquisition type 3D cartesian Acquired resolution (mm3) 0.48 × 0.48 × 0.98 Reconstructed resolution (mm3) 0.48 × 0.48 × 0.48 Field of view (mm3) 339 × 339 × 75 Flip angle 25° TR/TE (ms) 4.23/1.36 Nominal scan time (min:sec) 0:55 |
| 4 | Siemens Skyra (3 Tesla) | Gadovist | Flowrate of 2 ml/s, succeeded by a 25-ml saline flush | CE-MRA Slice orientation Coronal Acquisition type 3D cartesian Acquired resolution (mm3) 0.8 × 0.8 × 8 Reconstructed resolution (mm3) 0.8 × 0.8 × 0.8 Field of view (mm3) 340 × 275 × 70 Flip angle 25° TR/TE (ms) 3.37/1.36 Nominal scan time (min:sec) 0:19 |
| 5 | Siemens Vida (3 Tesla) | Gadovist | Flowrate 1,5-3ml/s, succeeded by a 25-ml saline flush | CE-MRA Slice orientation Coronal Acquisition type 3D cartesian Acquired resolution (mm3) 0.8 × 0.8 × 8 Reconstructed resolution (mm3) 0.8 × 0.8 × 0.8 Field of view (mm3) 340 × 275 × 70 Flip angle 25° TR/TE (ms) 3.37/1.36 Nominal scan time (min:sec) 0:19 |

**Table S2.**Diagnostic Accuracy Measures to Differentiate between Pseudo-Occlusion or True Occlusion of the Cervical Internal Carotid Artery stratified by each Individual Rater.

| Rater | Sensitivity (%) | | Specificity (%) | Accuracy (%) |
| --- | --- | --- | --- | --- |
| Fellow 1 | 75 (48-93) | 76 (55-91) | | 76 (60-88) |
| Fellow 2 | 69 (41-89) | 72 (51-88) | | 71 (54-84) |
| Fellow 3 | 44 (20-70) | 92 (74-99) | | 73 (57-86) |
| Board-certified radiologist 1 | 88 (62-98) | 84 (64-95) | | 85 (71-94) |
| Board-certified radiologist 2 | 75 (48-93) | 96 (80-99) | | 88 (74-96) |
| Board-certified radiologist 3 | 81 (54-96) | 96 (80-99) | | 90 (77-97) |

Data in parentheses indicate 95% confidence intervals.
